# Effects of sulphur amino acids on the size and structure of microbial communities of aerobic granular sludge bioreactors

**DOI:** 10.1007/s00726-022-03168-y

**Published:** 2022-05-25

**Authors:** Aurora Rosa-Masegosa, Lizandra Perez-Bou, Barbara Muñoz-Palazon, Antonio Monteoliva-García, Alejandro Gonzalez-Martinez, Jesus Gonzalez-Lopez, David Correa-Galeote

**Affiliations:** 1 Microbiology Department, Faculty of Pharmacy, University of Granada, Andalucía, 18071 Granada, Spain; 2 Microbiology and Environmental Technology Section, Institute of Water Research, University of Granada, Andalucía, 18071 Granada, Spain; 3 Microbial Biotechnology Group, Microbiology and Virology Department, Faculty of Biology, University of Habana, Habana, Cuba; 4 Department of Civil Engineering, University of Granada, 18071 Granada, Spain

**Keywords:** Cysteine, Methionine, qPCR, Illumina sequencing, N-removal, AGS-SBR

## Abstract

Granular activated sludge has been described as a promising tool in treating wastewater. However, the effect of high concentrations of sulphur amino acids, cysteine and methionine, in the evolution, development and stability of AGS-SBRs (aerobic granular sludge in sequential batch reactors) and their microbial communities is not well-established. Therefore, this study aimed to evaluate microbial communities' size, structure and dynamics in two AGS-SBRs fed with two different concentrations of amino acids (50 and 100 mg L^−1^ of both amino acids). In addition, the impact of the higher level of amino acids was also determined under an acclimatization or shock strategy. While N removal efficiency decreased with amino acids, the removal of the organic matter was generally satisfactory. Moreover, the abrupt presence of both amino acids reduced even further the removal performance of N, whereas under progressive adaptation, the removal yield was higher. Besides, excellent removal rates of cysteine and methionine elimination were found, in all stages below 80% of the influent values. Generally considered, the addition of amino acids weakly impacts the microbial communities' total abundances. On the contrary, the presence of amino acids sharply modulated the dominant bacterial structures. Furthermore, the highest amino acid concentration under the shock strategy resulted in a severe change in the structure of the microbial community. *Acidovorax*, *Flavobacterium*, *Methylophilus*, *Stenotrophomonas* and *Thauera* stood out as the prominent bacteria to cope with the high presence of cysteine and methionine. Hence, the AGS-SBR technology is valuable for treating influents enriched in sulphur Aa inclusively when a shock strategy was used.

**Supplementary Information:**

The online version contains supplementary material available at 10.1007/s00726-022-03168-y.

## Introduction

The global population is currently increasing exponentially, and it is expected to rise around 30% in the following years, reaching 9.9 billion people by 2050 (Bahar et al. 2020). In addition, many of the essential human activities, such as crop and livestock production, manufacturing of goods, power generation, or domestic activities, rely upon the availability of water in both adequate quantities and of acceptable quality (Huang et al. 2018). Therefore, the increase in the global population is coupled in parallel with higher water requirements (Jones et al. 2021). Concomitantly, sustainable water management is imperative to ensure water availability and easy access worldwide (Mole and Brooks, 2019). On the other hand, the increase in water requirements resulted in the generation of higher quantities of wastewater (WW), making its management a major concern for people's health. Also, it is an environmental hazard due to the ineffective and inefficient operations of WW collection and management to generate high-quality effluents (Zhang et al. 2019).

According to these reasons and to ensure water safety, it is crucial to design and develop integrated WW treatment systems that tackle environmental, social and economic concerns (Luqman and Al-Ansari, 2021; Derakhshan et al.2018a,b). In this regard, different compact and efficient biological WW treatment technologies have been designed during the past years such as membrane photobioreactors or aerobic granular sludge (AGS) (Derakhshan et al. 2018a,b) technology. The AGS system has turned into a promising tool over other biological methods due to different advantages as follows: excellent settling properties, high biomass retention, low production of sludge excess, removal nutrients and carbon in the same chamber and capacity to operate at shorter hydraulic retention times (Adav et al. 2008; Nancharaiah and Reddy, 2018; Rosa-Masegosa et al. 2021). Additionally, secondary settlers are unnecessary (Wilén et al. 2018). Therefore, AGS technology presents a 20–25% reduction in operation cost, 23–40% fewer electricity needs and a 50–75% reduction in space requirements because of the absence of secondary settlers (de Sousa Rollemberg et al. 2018). The AGS technology is usually applied in sequential batch reactors (Sun et al. 2019). Aerobic granules are strong and compact microbial biofilm structures in which different microorganisms form spherical aggregates, which have specific functions for removing organic material, nitrogen, phosphorus and recalcitrant compounds due to the presence of aerobic, anoxic and anaerobic microenvironments (He et al. 2016; Winkler et al. 2018; Castellanos et al. 2021). Hence, AGS-SBR has been greater spread due to their associated advantages over other conventional or emerging technologies (Bengtsson et al. 2019).

Another major challenge concerning the high global population growth rate is providing the food demand in an environmentally sustainable way (Prosekov and Ivanova, 2018). In this regard, meat consumption has risen, driven by increasing average individual incomes and population growth (Godfray et al. 2018). Nowadays, animal products represent around 50% of protein intake for humans (White and Hall, 2017), and it has been projected to increase by 75–80% by 2050 (Ritchie et al. 2018). Given the challenge of providing adequate nutrition for a growing global population, a corresponding increase in livestock farms is necessary. However, the vast amounts of WW generated from this sector and their high strength, especially the high level of nitrogen (N), have turned their treatment into one of the most critical environmental issues in recent years (Nagarajan et al. 2019). For example, pig farms generate 4–8 L WW per day and pig, amounting to 16 kg N per animal and year (García et al. 2017). In addition, when dealing with N-rich animal WW, their treatment process inevitably encounters amino acids (Aa) buildup due to proteins decomposition (Chen et al. 2019). Single Aa are metabolized by several biochemical processes, mainly hydrolysis, decomposing, acetogenesis and methanogenesis (Liu et al. 2019a). These catabolic reactions generate high concentrations of NH_4_^+^, which inevitably results in an increased presence of free ammonia (FA, NH_3_), inhibiting different key microorganisms involved in WW treatment (Park et al. 2014, 2015). Moreover, the presence of single Aa can act as a selection pressure inducing changes in the behaviour of microbial communities decreasing specific microbial activities and even modulating the size and structure of the microbial communities (Wang et al. 2017). In this sense, ammonia-oxidizing bacteria (AOB), denitrifiers, phosphate-accumulating organisms (PAO) or methanogens could be inhibited by a high level of single Aa (Gonzalez-Martinez et al. 2016; Rodriguez-Sanchez et al. 2016; Liu et al. 2019a). Besides, the sulphur Aa cysteine and methionine are highlighted by their lower removal rates in WW, resulting in their persistence in the effluents. Hence, according to Park et al. (2014), high levels of sulphur Aa may reduce the efficiency of WW treatment.

Despite the advantages of AGS over conventional WW technologies, AGS-SBR systems have been scarcely applied to treat WW enriched in Aa (Tang et al. 2021). Subsequently, there is a large knowledge gap in the effect of high levels of Aa in sewage treatment using AGS technology. Furthermore, the impact of the presence of the persistent sulphur Aa in the granule's microbial community technology is not well-established. Hence, to better understand the effects of high levels of single Aa on AGS-SBR, a better characterization of the size and structure of microbial populations of AGS subjected to the presence of amino acid-enriched influents is necessary.

Therefore, this study aimed to investigate the size and structure of the microbial communities of two AGS-SBR receiving influents enriched in cysteine and methionine, using quantitative PCR (qPCR) and high-throughput Illumina Miseq sequencing, respectively. Furthermore, the dynamics of the microbial communities subjected to a different level of sulphur Aa applied under acclimatization or a shock strategy were addressed. Finally, the evolution, development and stability of AGS-SBRs, the nutrient removal and the Aa elimination ratio were linked to the microbial communities.

## Materials and methods

### Bioreactors setup and operation

Two cylindrical column reactors at lab-scale were operated as AGS under sequential batch cycles for (AGS-SBR), which were used to treat synthetic wastewater enriched in cysteine and methionine.

The bioreactors were inoculated with 1 L of mature granules previously cultivated at a lab-scale in the Water Research Institute (University of Granada). After inoculation, the SBR were fed with a synthetic medium simulating WW. The detailed composition of the synthetic WW used was as follows: CH_3_COONa 0.75 g·L^−1^, NH_4_Cl 0.25 g·L^−1^, MgSO_4·_7H_2_O 0.1 g·L^−1^, K_2_HPO_4_ 0.085 g·L^−1^, KCl 0.04 g·L^−1^, and KH_2_PO_4_ 0.03 g·L^−1^. The AGS-SBRs were kept at room temperature (14–17 °C), and the pH of the influent was maintained at values close to neutrality (7.8 ± 0.3). The air was diffused from the bottom using fine bubbles with a flow rate of 2 L·min^−1^, and the Dissolved Oxygen (DO) measured was close to saturation.

To compare the effect of Aa in the evolution, development and stability of AGS-SBRs and their microbial communities, two concentrations of cysteine and methionine (50 and 100 mg·L, respectively, for each Aa) were added for acclimatization bioreactor (AB) or a shock bioreactor (SB) in order to carry out the most optimal strategy for treating Aa. Operational conditions are shown in Table 1. In this sense, both bioreactors were first operated in an adaptation period until they reached a stable operating performance day 21 and 12 for AB and SB for Stage I, respectively (AB-ST-I and SB-ST-I). Lately, the influents of the bioreactors were amended with concentrations of 50 mg·L^−1^ of both amino acids,until day 59 and 43 for AB and SB for low concentration (LC) of Aa, respectively (AB-LC and SB-LC periods). However, two different strategies were followed before the feeding with the highest level of amino acids (100 mg·L^−1^) denominated high concentration (HC) stage. The AB was fed with the highest level of Aa (100 mg·L^−1^ of each) automatically applied after the 50 mg·L^−1^ stage, then the granular biomass was previously in contact with Aa and acclimatized to 50 mg L^−1^ of cysteine and methionine (until day 90, AB-HC). Following the opposite strategy, the SB was reinoculated with granular biomass, and it was operated without amino acids until reaching the steady state (days 44 – 59, SB-ST-II). Finally, the SB was fed with 100 mg·L^−1^ of both Aa until the end of the experiment (SB-HC stage) to determine the effect of the direct addition of 100 mg·L^−1^ of cysteine and methionine in granules no previously exposed to a high level of Aa.

**Table 1:** Design of bioreactor, start-up strategy and operational conditions

AGS-SBR design	Acclimatization bioreactor (AB)	Shock bioreactor (SB)
Cylindrical columns	Height-90 cm Diameter-7 cm	Height-75 cm Diameter-11.5 cm
Volume exchange per cycle	60%	60%
HRT	6 h	6 h

### Physico-chemical determination analysis

Samples of influent and effluent were taken periodically to determine the removal performance of organic matter through analysis of chemical oxygen demand (COD) and biological oxygen demand at day 5 (BOD_5_) according to the standard protocols described by APHA (2005). In addition, ammonium (NH_4_^+^), nitrate (NO_3_^−^), nitrite (NO_2_^−^) and phosphate (PO_4_^3−^) were measured using a Metrohm Ion Chromatograph to monitor the ability to remove nutrients, for those samples were measured in triplicate. For ammonium concentration was employed a Metrosep C2-150 cation column. While, an anion column, MetrosepA supp-4–250, was used to measure nitrite, nitrate and phosphate concentrations. Calibration curves were developed with the following concentrations: 1, 10, 100, 400, 700 and 1000 mg L-1 for NH_4_+ and 1, 10, 50, 100 and 300 mg L-1 for NO_2_^−^, NO_3_^−^, and PO_4_^3−^ (Muñoz-Palazon et al. 2021). The mixed liquor suspended solids (MLSS) on the bioreactors was determined following APHA (2005). The granules were monitored by measuring the size and settling ability periodically. For size determination, a scalimeter was used, while the settling velocity was measured using a 40-cm column and a chronometer (Muñoz-Palazon et al. 2021). All determinations were done in triplicate.

### Amino acids quantification

In order to determine the removal performance of cysteine and methionine of each bioreactor, the concentration of these Aa in influent and effluent samples was measured regularly, following the protocol described by Inoue et al. 2016 with slight modifications. Influent and effluent samples were pre-treated and filtered using PES filter with 0.22 μm of pore size. To avoid the equipment saturation, several dilutions were prepared in vials for the high-performance liquid chromatography (HPLC) injection in a volume of 1.5 mL. 12 µL were taken for influent samples of 50 mg·L^−1^; 6 μL for influent samples of 100 mg·L^−1^, while for effluent samples were taken 40 µL. The calibration curve was prepared using seven measurements from 10 to 500 μg·L^−1^. The analyses were performed on a 1260 Infinity II LC System (Agilent, USA) with triple quadrupole. Cysteine and methionine were measured in triplicates.

The mobile phase composition was obtained on an InfinityLab Poroshell120 EC-C18 column (Agilent) with 0.1% formic acid in water and 0.1% formic acid in acetonitrile (ACN). The HPLC analysis was performed by isocratic elution with a flow rate 0.4 mL·min^−1^ for 3 min.

### DNA extraction

Granules samples from the AGS-SBR reactors were used to perform a microbial characterization. In this regard, 0.5 g of biomass was used for the isolation of total deoxyribonucleic acid (DNA) using the FastDNA SPIN Kit and the FastPrep 24-Instrument (MP Biomedicals, Germany), according to the 'manufacturer's protocol. Two independent extractions were made at each sampling time.

### Quantifications of total populations and functional marker genes

Quantifications of total populations and functional marker genes were performed by quantitative PCR (qPCR) on a QuantStudio-3 Real-Time PCR system (Applied Biosystems). Gene quantification (2 independent experiments, 3 replicates per DNA sample) targeting the bacterial and archaeal 16S rRNA and fungal 18S rRNA genes were used as proxies for total *Bacteria*, *Archaea* and *Fungi*, respectively. The ammonia monooxygenase (*amoA*) gene was amplified to determine abundances of ammonia-oxidizing bacteria (AOB) and archaea (AOA) owing to that the limiting step in biological nitrogen removal is the oxidation of NH_4_^+^ to NO_2_^−^ (Limpiyakorn et al. 2013). Similarly, the N_2_O reductase (*nosZ*) gene was selected as a marker for denitrifiers since this gene codifies the last enzyme in the sequential reduction of NO_3_^−^ to N_2_ (Henry et al. 2006). Finally, 16S rRNA of *Candidatus* Accumulibacter and *Candidatus* Competibacter (hereunder referred to as *Accumulibacter* and *Competibacter*) were used as markers for polyphosphate-accumulating organisms (PAOs) and glycogen-accumulating organisms (GAOs), respectively. For the determination of the bacteria able to realize the dissimilatory NO_3_^−^ reduction, a TOPO^®^ TA plasmid carrying a 269-basepair of the *nrfA* gene from *E. coli* strain GY36 was constructed with the aid of the primers nrfAF2aw (5′-CAR TGY CAY GTB GAR TA) and nrfAR1 (5′-TWN GGC ATR TGR CAR TC) described by Welsh et al. (2014). All reaction mixtures were made in a total volume of 25 mL following Correa-Galeote et al. (2021a). Primer sequences and PCR conditions are provided in Table SI.1 in the Supplementary Material.

### Bacterial 16S rRNA gene high-throughput sequencing

Illumina sequencing was made using the primers Pro341F/Pro805R (Takahashi et al. 2014) to determine the structure of *Bacteria* and *Fungi* communities, respectively. The 16S rRNA bacterial data from next-generating sequencing was analyzed using Mothur V1.44.3 software (Schloss, 2016). Default settings were used for quality control, primer trimming, filtering, pre-clustering and chimera detection. Operational taxonomic units (OTUs) assigned at the 97% cut-off level with a relative abundance (RA) > 0.0001% were classified taxonomically using the 16S ribosomal database from the NCBI with the aid of the blast tool of Geneious Prime v.2019 software (Geneious, USA). Correa-Galeote et al. (2021b) describe the bioinformatics pipeline's full details. The 16S rRNA sequences retrieved were deposited in GeneBank under the accession numbers XXXX. Simpson and Shannon biodiversity indices were calculated according to Hill et al. (2003).

### Statistical analysis

The statistical differences of the different data sets among the samples were analyzed using the non-parametric Mann–Whitney and Kruskal–Wallis tests (*p* < 0.05 significance level) in XLSTAT v2020 (Addinsoft, USA). Heatmaps of the RAs of the dominant bacterial and fungal OTUs were made using the average clustering method of the pheatmap package in R studio v.3.4.1 (Rstudio, USA). The PC‐ORD software (Wild Blueberry Media, USA) was used to perform a nonmultimetric multidimensional scaling (NMS) analysis. Finally, the abiotic data sets were coupled into the NMS biplot space to obtain correlations among samples' ordination and their corresponding physicochemical parameters (Egghe and Leydesdorff, 2009).

## Results and discussion

### Granular properties

The granular properties (mean size and settle velocity) are shown in Fig. SI.1 and SI.2.

The height–diameter ratios of both AGS-SBR were smaller, and consequently, the granules were larger than in other studies (Muñoz-Palazon et al. 2021). The average size for the start-up oscillated from 7.4 to 15.7 mm during both steady-state periods (AB-ST-I and SB-ST-I). Then, the treatment of amino acids at low concentration (LC) (50 mg·L^−1^ for cysteine and 50 mg·L^−1^ methionine) affected notably to granules operated in SB with the smallest registered values of 10.3 mm, while higher Aa concentrations (HC) (100 mg·L^−1^ for cysteine and 10 mg·L^−1^ methionine) triggered a more deeply reduction of mean size in the SB, decreasing to values around 6 mm (SB-LC).On the contrary, AB reflected slight oscillations without any clear pattern for all the experiment. Consequently, more stability was observed in systems with progressive adaptation to high concentration of Aa, owing to that the biomass changes were less marked.

The settling velocity, one of the most relevant advantages of AGS technology (de Sousa Rollemberg et al. 2018), followed the same trend that the mean size, because of the strong drop in terms of speed, approximately a 50% reduction (82.6 m h^−1^ to 47.5 m h^−1^), for LC-SB and HC-SB). Oppositely, the settling ability in AB was improved over operational time, although some affections were denoted in the AB-HC stage. In addition, the inverse relation of mean size and settling velocity of AB-HC pointed out that granules were denser and smaller when confronted with Aa, and as a consequence the decantation ability improved, as reflected the end of the AB-HC-II.

Mix liquor suspended solids (MLSS).

During the start-up, each reactor achieves a stable concentration of biomass. In the case of AB, this value was close to 3 g·L^−1^, while for SB was 4.3 g·L^−1^ (Fig. SI.3) After the addition of 50 mg·L^−1^ of each amino acid (SB-LC), SB suffered a deep reduction of MLSS, with values below 2 mg·L^−1^. For AB, a progressive increment of MLSS was observed until day 28 (AB-ST-I), in fact as consequence of biomass growth, the sludge excess was discarded on day 15. Next, the biomass concentration decreased and was stabilized in values ranging 2 to 3 mg·L^−1^, whose values remained in the next stage (AB-HC). On the other hand, the treatment of Aa-containing WW by AGS in shock scenario was turned out to the breakage of granules and over wash-out of biomass, with values below 2 mg·L^−1^. Later, the SB was inoculated again (phase SB-ST-II) and it was operated until reach a stable concentration of biomass, rounding 3 g·L^−1^. After the supplementation of 100 mg·L^−1^ of methionine and 100 mg·L^−1^ of cysteine was done, reactor suffered a robust shrinkage of MLSS, with values below 1.5 g·L^−1^, even it was registered values close to 0.5 g·L^−1^ at the end of HC stage. In summary, SB experimented a reduction of MLSS with the addition of Aa, while AB suffered oscillation in terms of MLSS, but after a short period of adaptation to the influent with Aa, it reached to mean values similar to those initially obtained. These results denoted and corroborated that the acclimatization processes promoted a better performance of AGS for treating Aa-containing WW in terms of concentration of biomass, while reactors without previous adaptation treating HC of Aa had remarkable adverse effects related to MLSS. This pattern was similar to that found in the nitrogen removal ratio’ therefore, we could conclude that AGS subjected to progressive and gradual adaptation processes improves the yields obtained, mainly pointing out the capacity to retain biomass and the removal of nutrients.

### Physico-chemical evaluation of AB and SB

**Fig. 1:**
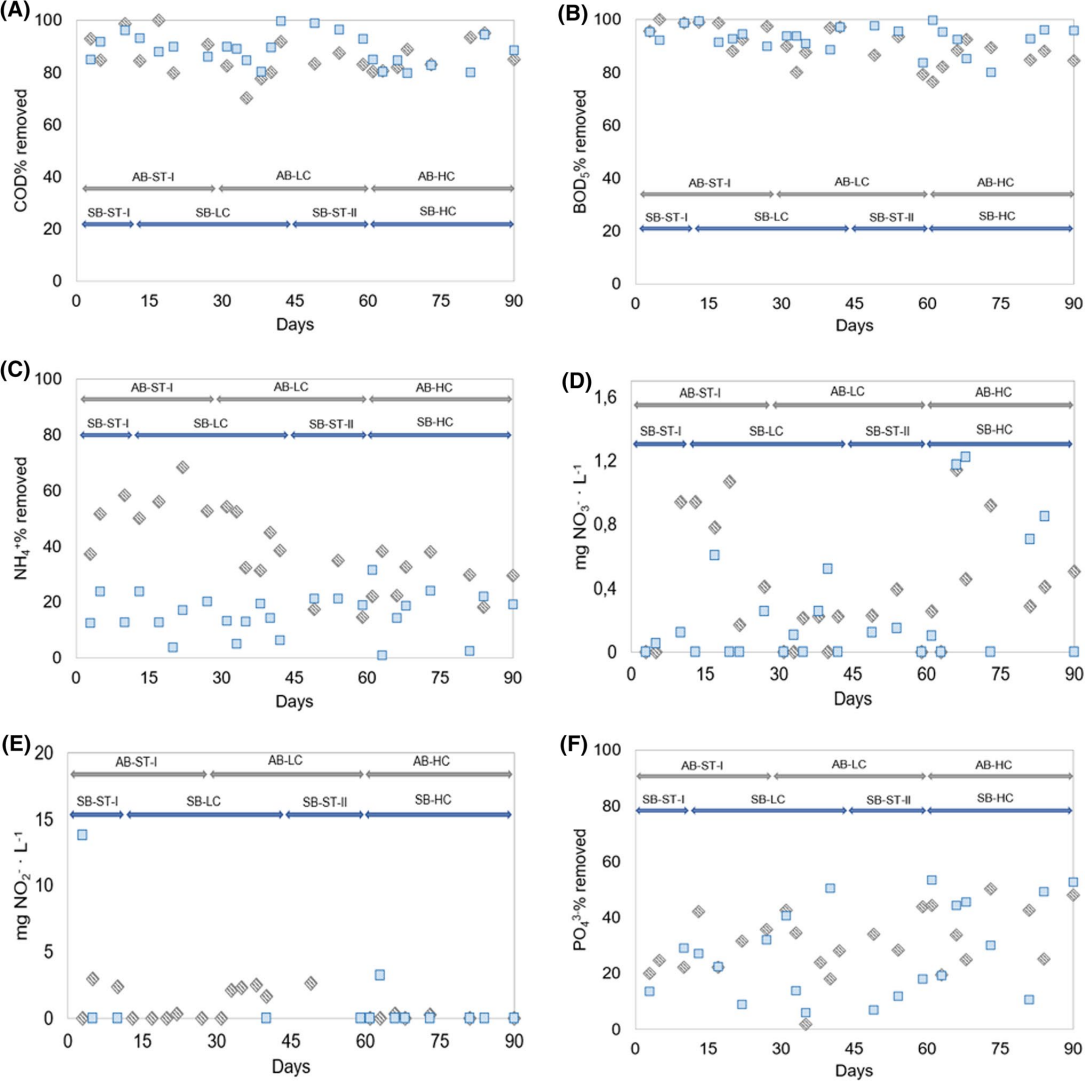
Chemical determinations of AGS-SBRs operated using different concentrations of cysteine and methionine under an acclimatization (*AB* grey diamonds) or shock strategy (*SB* blue squares) for COD removal ratio (**A**); BOD5 removal ratio (**B**); ammonia removal ratio (**C**); nitrate removal ratio (**D**);phosphate removal ratio (**E**)

COD removal ratio for both bioreactors is shown in the Fig. 1A. On the one hand, the SB system during the ST-I reached values until 96.2% of total COD removal. Therefore, when the influent was characterized by the presence of low concentration of Aa it had slight oscillations with reduction of the performance, but never below 80% of COD removal, although at the end of this stage the systems reflected an adaptation because the values were recovered with values close to 100%. Then, the system was inoculated again with granules without previous contact with Aa (SB-ST-II), and the results were successful because they were never below 80%. Moreover, it was possible to observe at the beginning of the HC stage that the average removal ratio was 82%, while after an adaptation period, the COD removal ratio achieved more than 91% of average percent. Thus, it could be concluded that when granular biomass is treating amino acids, the COD performance not induce to graves consequences derived from influent characteristics. On the other hand, the AB-ST-I demonstrated high capacity to degrade COD (with values ranging from 80 to 100%) like SB-ST-I and II. When the system started to treat influent containing Aa, detrimental effects were observed, over all at operational day 35, when the AB-LC only achieve to degrade of 70% of COD from the influent, although as occurred in SB, after adaption of granules to the amino acids presence, the performance was improved. These granular biomass was subsequently subjected to HC of amino acids and the results were excellent. Thus, previous granular biomass adaptation to the amino acids promote a good removal ratio as reflected by the trend in AB-HC.

Biodegradable organic matter removal analyzed by BOD_5_ for AB and SB achieved a good efficiency (Fig. 1B). The tendency was almost the same that COD removal, although the degradation ratio was almost 10 points higher than in COD. Few operational days of SB and AC reflected adverse consequences possibly caused by the inhibition of bacterial metabolic pathways that conform the granules, facts that Liu et al. (2019b) also reported. For AB-LC, at operational day 35, BOD_5_ removal ratio suffered a depletion, these values noticed the damage produce to heterotrophic microorganisms within granule as consequence of Aa presence. In addition, the same pattern was recognized during the first days of operation in AB-HC. For SB-HC, the performance was mainly affected by the shock exerted by the high concentration of Aa contained in the influent synthetic wastewater, because BOD_5_ removal was decreasing until operational day 75, when the biological system retrieved the capacity to biodegrade organic matter.

Changes in the efficiency in terms of ammonia and nitrate concentrations under different concentrations of Aa (LC and HC) had strong repercussions on the performance of nutrient removal. The absence and/or inhibition of nitrogen metabolisms is an important issue linked with the Aa treatment, as reported Park et al. 2014; Gonzalez-Martinez et al. 2016; Rodriguez-Sanchez et al. 2016. Thus, the ammonia oxidation as well as denitrification process should be an important point of view of this study. In terms of ammonium oxidation capability, granular sludge operated in AB suffered deeply from the presence of cysteine and methionine in LC and HC stages, decreasing close to 60%, (70 mg NH_4_^+^·L^−1^). As general pattern, for AB, the oxidation was declining during operational time, marking day 25 as turning point (Fig. 1C). For SB, the ammonia oxidation was low even in LC stage. In fact, the highest removal ratio reached by SB-HC was 31% of total ammonia influent, while in AB- HC, the values were close to 40% of ammonia removal ratio. Despite that there was a clear effect of Aa on the ammonia oxidation, the scenario for shock bioreactor was negatively overwhelming. These results corroborate the results described by other authors, reporting the inhibition of ammonia oxidation metabolisms by the presence of cysteine and methionine (Park et al. 2014; Rodriguez-Sanchez et al. 2016). On the other hand, the nitrate concentration in AB effluent was at the maximum point of 1.14 mg·L^−1^, while results usually were in the range 0.2 to 0.4 mg NO_3_^−^·L^−1^ (Fig. 1D). The shock bioreactor showed a more accused trend, but their average values were always below 1.25 mg·L^−1^. Finally, no nitrite accumulation was observed during the whole experimentation in any of both reactors (Fig. 1E), so there was no inhibition of nitratation, since all oxidized ammonium was transformed to nitrate. This fact represents an advantage over other previously described systems (Rodriguez-Sanchez et al. 2016). These results suggested high presence of Aa affect to the nitritation process but not the nitratation process.

The phosphate accumulation by granular biomass was measured by ion chromatography. The phosphate removal capacity for AB and SB reactors in LC and HC stages are shown in the Fig. 1F. Certainly, at the beginning of AB-HC stage, a detriment in phosphate removal was denoted, but it was recovery over operational time, achieving values close to 50%. Similarly, the P removal of SB followed the same behaviour described for AB, but with a trend more pronounced. In addition, oscillations were found without following clear pattern. Then, it could be suggested that Aa had adverse effects on the phosphate accumulation and removal by AGS. The optimal conditions for phosphate removal by biological process required anoxic and aerobic niches, then the partial breakage of granules in SB-HC promoted the absence of niches in limited oxygen concentration by the reduction of granular cores. Moreover, Quoc et al. (2021) pointed out as higher particle sizes had lower phosphate uptake ratio.

### Amino acid removal rate

**Fig. 2:**
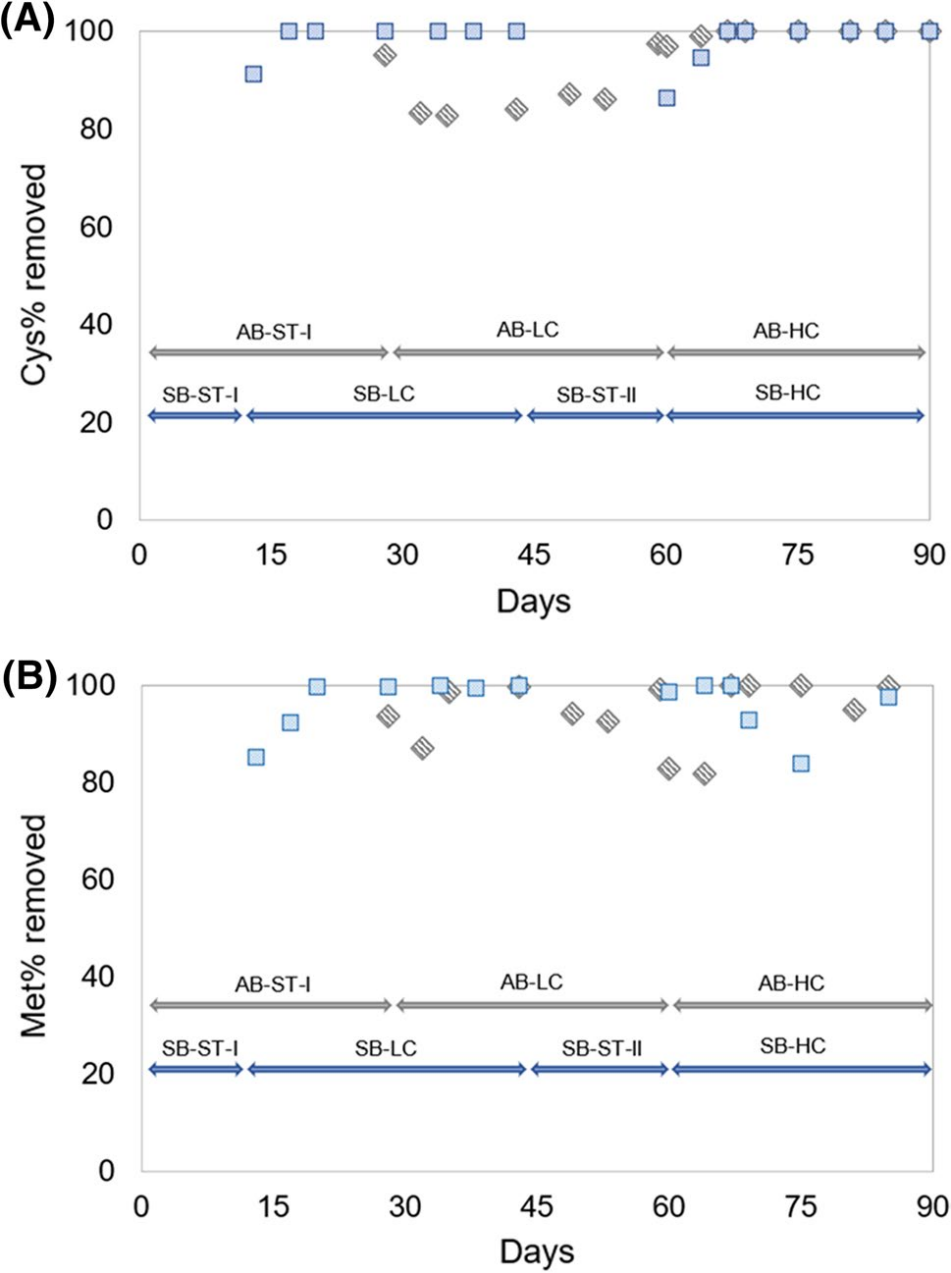
Cysteine (**A**) and methionine (**B**) removal ratio for both AGS-SBRs operated using different concentrations of amino acids under an acclimatization (*AB* grey diamonds) or shock strategy (*SB* blue squares). *Cys* cysteine, *Met* methionine

In general terms, a high Aa removal rate was observed for both reactors, above 80% of degradation (Fig. 2). However, several obvious differences were found among periods and reactors. On the one hand, SB had excellent removal rate for cysteine (Fig. 2A), since in a short period it reached a 100% of degradation for both LC and HC stages. It was noted that the stabilization period for SB-HC took longer time than for SB-LC stage, because in HC it was needed 7 days in comparison to SB-LC, when only 4 days were necessary to achieve the complete removal. On the other hand, for AB, the removal rate of cysteine in AB-LC stage was lower than for SB-LC, with values above 80%. Contrarily, in AB-HC stage a total Aa degradation was quickly achieved after9 days of operation under these conditions, following the same trend of SB treating Aa-high-containing WW. For methionine, SB-LC followed a similar tendency than for cysteine, in which 100% of removal ratio was achieved. This fact demonstrated the high capability of AGS technology to transform and degrade Aa. In SB-HC stage, removal ratio reached was close to 100%, this fact could be encouraged by bio-adsorption processes, given the characteristics of granular surface, because after 15 days of operation in SB-HC stage, the removal ability suffered a progressive decrease in terms of degradation, trend that changed after several days of operation, as shown Fig. 2B. Then, this result could suggest that the granules were not able to remove methionine efficiently as at the beginning of the phase. This could possibly be due to the the decreasing of MLSS and by the release of Aa driven by desorption processes. Bioadsorption–desorption process was suggested because after a period with lower efficiencies, the removal rate was recovered until it reached 100% at the end of the experiment, despite low biomass concentration. As for cysteine, AB-LC had a lower removal ratio of methionine than SB-LC. In this regard, AB-LC stage started with values around 90% of degradation, but at the end of that period the degradation increased with values ranging from 94.0 to 99.6%. However, in AB-HC, the degration of methionine was highly oscillating as previously described for SB-HC. Therefore, clear differences were observed in the degradation patterns of both Aa, methionine being more persistent than cysteine in the WW treatment. Also, there is to note the excellent removal rates of both Aa using an AGS-SBR technology independently of the feeding strategy.

### Quantification of bacterial, archaeal and fungal populations

**Fig. 3:**
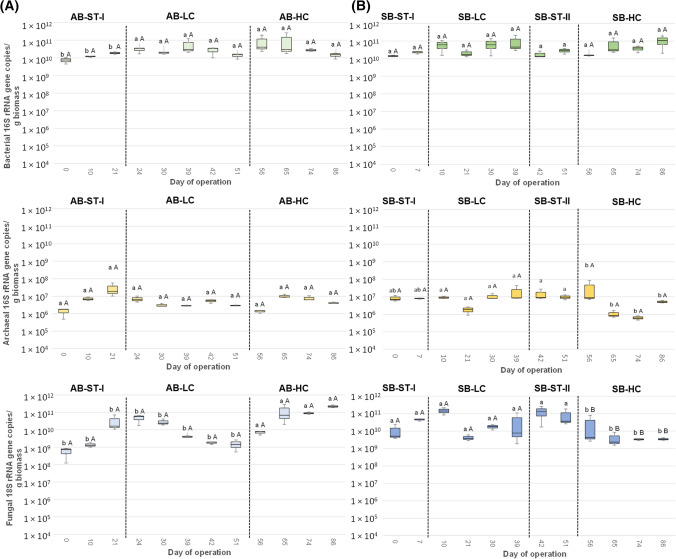
Total abundances of bacterial and archaeal 16S rRNA, fungal 18S rRNA determined as the gene copies g-1 biomass by quantitative PCR (*n* = 6) retrieved from **A** acclimatization (*AB*) and **B** shock (*SB*) AGS-SBRs. Lowercase letters indicate significant differences among periods for a given reactor according to the Kruskal–Wallis and Conover–Iman tests (*p* < 0.05). Capital letters indicate significant differences between periods AB-ST-I and SB-ST-I, AB-LC and SB-LC, and AB-HC and SB-HC according to the Mann–Whitney (*p* < 0.05)

The total gene copies of the *Bacteria*, *Archaea* and *Fungi* are summarized in Fig. 3.

The total bacterial populations were in the range of 7.47 × 10^9^–1.04 × 10^11^ and 1.42 × 10^10^–1.06 × 10^11^ gene copies g^−1^ of biomass for AB and SB, respectively. According to the Kruskal–Wallis test, no statistical differences between operational periods in any AGS-SBR nor between reactors for a given period were determined. Therefore, none of the concentrations of Aa (50 or 100 mg L of cysteine and 50 or 100 mg L of methionine) had a stronger effect on the total bacterial populations under the different feeding strategies tested. The total quantity of *Bacteria* has been proposed as a general marker of feature WW treatment, wherein several organic compounds are mineralized to H_2_O and CO_2_ by bacteria (Martinez-Alcala et al. 2017). Hence, the nutrient removal efficiency was not inhibited by the high presence of both Aa as it was depicted from the BOD_5_ data (Fig. 1B).

Regarding the abundance of total *Archaea*, independently of the sample, the genes copies of *Archaea* population were lower than those of *Bacteria* (ranging from 6.30 × 10^5^ and 3.35 × 10^7^ gene copies g^−1^ of biomass) in agreement with previous studies that reported this fact (Gonzalez-Martinez et al. 2018; Muñoz-Palazon et al. 2018). For the AB reactor, there were no differences among periods. However, the addition of 100 mg L^−1^ of Aa reduced the archaeal populations in SB-HC. Interestingly, no differences were determined between reactors in any of the three periods compared.

Finally, the fungal populations were in the range of 5.67 × 10^8^–2.20 × 10^11^ and 3.32 × 10^9^–1.49 × 10^11^ for AB and SB, respectively. Regarding the differences among strategies, whereas the supply of 100 mg L^−1^ influent stimulated the fungal population in AB-HC compared with AB-LC, the shock strategy reduced the abundance of *Fungi* in SB. Also, lower fungal quantities were detected in SB-HC than in period AB-HC. Therefore, the high presence of Aa under a shock supplied is an inhibitor of the fungal populations. Although *Fungi* play several processes in wastewater treatment, such as detoxification, granulation, and organic matter biodegradation (Niu et al. 2017), organic matter removal was not drastically affected in SB-HC. This suggested that other microbial groups carried out the organic matter degradation in this stage.

Finally, despite the differences among reactors and periods for the *Bacteria*, *Archaea* and *Fungi* abundances, these populations were in the same range as the previously described in other AGS-SBRs (Amorim et al. 2018; Gonzalez-Martinez et al., 2018; Muñoz-Palazon et al., 2018, 2021).

### Quantification of the functional marker genes

The total abundance of AOB and AOA, dissimilatory nitrate-reducing and denitrifying bacteria, and PAO and GAO populations are shown in Table 2.

**Table 2:** Gene copies/g biomass of ammonia-oxidising bacteria and archaea, dissimilatory nitrate-reducing and denitrifying bacteria, and PAO and GAO populations determined by quantitative PCR (*n* = 6) retrieved from acclimatization (AB), and shock (SB) AGS-SBRs

Period	Day	Ammonia-oxidising bacteria	Ammonia-oxidising archaea	Dissimilatory nitrate-reducing bacteria	Denitrifying bacteria	PAO	GAO
AB-ST-I	0	1.59 × 10^4^ ± 1.09 × 10^4^ b A	bld a A	3.69 × 10^8^ ± 2.66 × 10^7^ a A	1.75 × 10^8^ ± 7.07 × 10^7^ a A	2.46 × 10^7^ ± 1.50 × 10^7^ a A	bld a A
	10	2.84 × 10^4^ ± 9.79 × 10^3^ b A	bld a A	1.17 × 10^9^ ± 2.65 × 10^8^ a A	4.96 × 10^8^ ± 1.90 × 10^8^ a A	1.56 × 10^8^ ± 3.58 × 10^7^ a A	bld a A
	21	2.46 × 10^5^ ± 1.10 × 10^5^ b A	4.19 × 10^4^ ± 8.62 × 10^3^ a A	6.01 × 10^9^ ± 3.64 × 10^8^ a A	5.73 × 10^8^ ± 1.22 × 10^8^ a A	1.22 × 10^9^ ± 7.00 × 10^8^ a A	4.15 × 10^6^ ± 2.94 × 10^6^ a A
AB-LC	24	1.16 × 10^5^ ± 6.37 × 10^4^ b A	1.27 × 10^4^ ± 3.14 × 10^3^ a A	3.33 × 10^9^ ± 4.11 × 10^8^ a A	2.17 × 10^8^ ± 5.52 × 10^7^ a B	1.40 × 10^9^ ± 6.42 × 108 a A	1.88 × 10^5^ ± 6.70 × 10^4^ a A
	30	6.22 × 10^4^ ± 3.21 × 10^4^ b A	bld a A	2.60 × 10^9^ ± 3.40 × 10^8^ a A	1.42 × 10^8^ ± 1.10 × 10^7^ a B	8.18 × 10^8^ ± 7.69 × 10^7^ a A	1.50 × 10^5^ ± 2.08 × 10^3^ a A
	39	5.11 × 10^4^ ± 1.33 × 10^4^ b A	bld a A	4.83 × 10^8^ ± 1.08 × 10^8^ a A	2.91 × 10^8^ ± 8.18 × 10^7^ a B	1.09 × 10^8^ ± 1.47 × 10^7^ a A	bld a A
	42	1.31 × 10^5^ ± 2.94 × ^104^ b A	1.44 × 10^4^ ± 7.43 × 10^3^ a A	1.21 × 10^9^ ± 3.72 × 10^8^ a A	8.68 × 10^8^ ± 5.82 × 10^8^ a B	3.39 × 10^8^ ± 1.14 × 10^8^ a A	6.21 × 10^5^ ± 4.10 × 10^5^ a A
	51	3.93 × 10^4^ ± 2.57 × 10^3^ b A	bld a A	5.27 × 10^8^ ± 1.28 × ^107^ a A	2.65 × 10^8^ ± 1.68 × 10^8^ a B	3.54 × 10^7^ ± 6.08 × ^106^ a A	bld a A
AB-HC	56	6.30 × 10^4^ ± 1.73 × 10^4^ a A	bld a A	6.83 × 10^8^ ± 1.27 × 10^8^ a B	2.59 × 10^8^ ± 1.05 × 10^8^ a A	4.28 × 10^7^ ± 3.37 × 10^6^ a B	bld a A
	65	1.31 × 10^5^ ± 4.37 × 10^4^ a A	1.27 × 10^4^ ± 2.30 × 10^3^ a A	1.05 × 10^9^ ± 3.10 × ^108^ a B	1.98 × 10^9^ ± 8.74 × 10^8^ a A	6.68 × 10^7^ ± 1.84 × 10^7^ a B	bld a A
	74	2.79 × 10^5^ ± 3.10 × 10^4^ a A	1.76 × 10^4^ ± 1.17 × 10^3^ a A	1.25 × 10^9^ ± 2.11 × ^108^ a B	7.53 × 10^8^ ± 1.32 × 10^8^ a A	1.49 × 10^8^ ± 1.96 × 10^7^ a B	bld a A
	86	1.62 × 10^5^ ± 9.26 × 10^4^ a A	3.72 × 10^4^ ± 3.97 × 10^3^ a A	5.81 × 10^9^ ± 9.18 × 10^8^ a B	2.24 × 10^8^ ± 4.21 × ^107^ a A	9.59 × 10^7^ ± 7.63 × 10^6^ a B	4.92 × 10^4^ ± 1.27 × 10^4^ a A
SB-ST-I	0	6.86 × 10^4^ ± 2.37 × 10^4^ a A	1.08 × 10^4^ ± 3.95 × 10^3^ b A	1.44 × 10^9^ ± 2.08 × 10^8^ a A	1.48 × 10^9^ ± 2.16 × 10^9^ c A	1.79 × 10^8^ ± 4.01 × 10^7^ a A	bld a A
	7	2.41 × 10^4^ ± 1.01 × 10^4^ a A	bld b A	3.94 × 10^9^ ± 3.87 × 10^8^ a A	2.96 × 10^8^ ± 8.31 × 10^7^ c A	2.01 × 10^8^ ± 1.50 × 10^8^ a A	7.26 × 10^4^ ± 1.68 × 10^4^ a A
SB-LC	10	6.61 × 10^4^ ± 3.30 × 10^4^ a A	1.51 × 10^5^ ± 1.39 × 10^5^ b A	1.94 × 10^10^ ± 7.75 × 10^8^ a A	3.77 × 10^8^ ± 1.18 × 10^7^ a A	2.00 × 10^9^ ± 6.49 × 10^8^ a A	1.45 × 10^6^ ± 5.54 × 10^5^ a A
	21	3.65 × 10^4^ ± 2.39 × 10^4^ a A	bld b A	4.65 × 10^8^ ± 1.54 × 10^8^ a A	1.78 × 10^9^ ± 4.25 × 10^8^ a A	5.70 × 10^7^ ± 2.48 × 10^7^ a A	bld a A
	30	6.24 × 10^4^ ± 3.34 × 10^4^ a A	1.47 × 10^4^ ± 1.46 × 10^3^ b A	1.21 × 10^9^ ± 3.46 × 10^8^ a A	1.06 × 10^9^ ± 1.03 × 10^8^ a A	8.49 × 10^7^ ± 1.85 × 10^7^ a A	bld a A
	39	2.24 × 10^5^ ± 1.11 × 10^5^ a A	1.28 × 10^4^ ± 1.97 × 10^3^ b A	9.00 × 10^9^ ± 4.69 × 10^8^ a A	2.03 × 10^9^ ± 8.98 × 10^8^ a A	2.81 × 10^8^ ± 9.58 × 10^7^ a A	2.28 × 10^6^ ± 1.02 × 10^6^ a A
SB-ST-II	42	5.53 × 10^4^ ± 1.21 × 10^4^ a	6.93 × 10^4^ ± 5.74 × 10^4^ a	6.96 × 10^9^ ± 4.33 × 10^8^ a	4.13 × 10^8^ ± 1.55 × 10^8^ bc	2.12 × 10^8^ ± 8.99 × 10^7^ a	4.58 × 10^6^ ± 4.14 × 10^6^ a
	51	9.41 × 10^4^ ± 6.56 × 10^4^ a	2.60 × 10^4^ ± 9.78 × 10^3^ a	4.04 × 10^9^ ± 5.30 × 10^8^ a	6.69 × 10^8^ ± 7.91 × 10^7^ bc	7.67 × 10^8^ ± 3.47 × 10^8^ a	bld a
SB-HC	56	3.76 × 10^4^ ± 6.74 × 10^3^ a B	1.27 × 10^4^ ± 4.17 × ^103^ a A	2.12 × 10^9^ ± 6.69 × 10^8^ a A	3.50 × 10^8^ ± 1.49 × 10^8^ ab A	2.82 × 10^8^ ± 4.68 × 10^7^ a A	bld a A
	65	5.46 × 10^4^ ± 3.26 × 10^4^ a B	2.81 × 10^4^ ± 1.72 × 10^3^ a A	1.01 × 10^10^ ± 8.71 × 10^8^ a A	1.49 × 10^9^ ± 7.59 × 10^8^ ab A	3.06 × 10^8^ ± 1.11 × 10^8^ a A	bld a A
	74	9.49 × 10^4^ ± 1.96 × 10^4^ a B	8.48 × 10^4^ ± 4.86 × 10^3^ a A	3.05 × 10^9^ ± 4.55 × 10^8^ a A	1.52 × 10^9^ ± 3.56 × 10^8^ ab A	6.11 × 10^8^ ± 1.84 × 10^8^ a A	1.74 × 10^5^ ± 1.82 × 10^4^ a A
	86	5.93 × 10^4^ ± 2.91 × 10^4^ a B	3.58 × 10^4^ ± 1.27 × 10^4^ a A	5.48 × 10^9^ ± 6.74 × 10^8^ a A	1.00 × 10^9^ ± 1.00 × 10^8^ ab A	3.56 × 10^9^ ± 1.31 × 10^9^ a A	2.45 × 10^5^ ± 3.43 × 10^4^ a A

According to the Kruskal–Wallis test, lowercase letters indicate significant differences among periods for a given reactor (*p* < 0.05). According to the Mann–Whitney (*p* < 0.05), capital letters indicate significant differences between periods AB-ST-I and SB-ST-I, AB-LC and SB-LC, and AB-HC and SB-HC. bld: below the detection limit (< 1.00 × 10^4^ copies/g biomass)

The abundance of bacterial *amoA* genes oscillated between 1.59 × 10^4^ to 2.70 × 10^5^ for AB and between 2.41 × 10^4^ to 2.24 × 10^5^ copies g^−1^ biomass for SB. Similarly, the abundance of AOA oscillated from below the detection limit (< 1.00 × 10^4^ copies g^−1^ biomass) to 4.19 × 10^4^ and 1.51 × 10^4^ copies/g biomass for AB and SB, respectively. Therefore, the dominance of AOB populations over AOA populations was found, suggesting a more relevant role of AOB over AOA in NH_4_^+^ removal in this study. There is to note that the high level of Aa had a different stimulative effect over the AOB populations in both reactors. In this sense, whereas the abundance of AOB was increased when the acclimatization strategy was implemented (AB-HC), AOB populations of SB did not significantly change among operational periods. In addition, a lower number of bacterial *amoA* genes was found in SB-HC compared with AB-HC. According to Gwak et al. (2020), the presence of complex organic compounds can reduce ammonium-oxidizing microorganisms. Therefore, the failure in NH_4_^+^ removal observed in SB-HC (Fig. 1) could be linked to the inhibition of AOB populations when the microbial population were not previously in contact with a high level of Aa. Despite the differences in the AOB populations, their ranges agreed with the described in other AGS-SBRs (Thwaites et al., 2017; Amorim et al., 2018).

Generally considered, the abundances of the *nosZ* gene were in the same range for both AGS-SBRs (1.42 × 10^8^ – 2.03 × 10^9^ copies g^−1^ biomass), and there were no differences in the denitrifying populations among periods in none reactors. The abundances of denitrifiers were in the same range as the described in other AGS-SBR (Muñoz-Palazon et al., 2020); however, a lower transformation of NO_3_^−^ was found in both reactors. This indicates that despite the high abundance of *nosZ*-bearing bacteria in the granule, the microorganism performing the last step of biological nitrogen removal (Wigginton et al., 2020) was not active enough to transform all NO_3_^−^ into N_2_ in both SBRs.

The abundance of *nrfA* gene oscillated between 3.69 × 10^8^ to 1.94 × 10^10^ gene copies g^−1^, independently of the reactor. Nevertheless, the gene copies of dissimilatory nitrate-reducing bacteria were more plentiful in period SB-HC than these AB-HC values. It is to be noted that dissimilatory nitrate reduction is a harmful process in wastewater N removal (Wang et al., 2020). Therefore, the over-abundance of the *nrfA* gene in SB-HC could aggravate the lower NH_4_^+^ removal rate found in this period due to the stimulative effect over dissimilatory nitrate-reducing bacteria of a high presence of sulphur Aa without a previous acclimatization period. To the best of the author's knowledge, this is the first study that evaluated the abundance of dissimilatory nitrate-reducing bacteria in AGR-SBRs; however, the gene copies of these populations agreed with the previously described in other wastewater treatment systems (Wang et al., 2020).

Finally, the quantity of 16S rRNA genes of *Accumulibacter* was between 2.46 × 10^7^ to 3.56 × 10^9^ gene copies g^−1^ biomass, and the abundance of *Competibacter* oscillated from below the detection limit to 4 4.58 × 10^6^ gene copies g^−1^ biomass independently of the SBR. Quantities of *Accumulibacer* and *Competibacter* felling within the previously described in other AGS-SBRs (Henriet et al., 2016; Wei et al., 2020). PAOs are the main microorganisms in P removal, whereas GAOs negatively impact the general WW treatment (Camejo et al., 2019). No differences in the abundances of both populations were observed among periods nor between reactors, suggesting stable populations of these populations independently of the concentration of Aa or the feeding strategy. However, phosphate removal did not reach successful results suggesting that PAO populations could not achieve the required level of efficacy.

### Determination of bacterial community dynamics

The number of high-quality sequences for the bacterial Illumina-sequencing was 2,049,864, and the average number per library was 42,706 ± 6980 sequences. Overall, 1382 different OTUs were found with an average number per amplicon library of 399 ± 52. The abundance of each OTU and their taxonomic classifications are shown in Table SI.2. In AB, the OTUs richness was statistically only reduced when the reactor was fed with 100 mg L^−1^ of Aa (AB-HC stage) compared with the remaining periods (Table SI.3). However, in SB, there was a reduction in the bacterial diversity in SB-LC and SB-HC, and higher and without differences in SB-ST-I and SB-ST-II. Therefore, the bacterial communities of both bioreactors subjected to a high level of Aa remarkably selected the bacterial communities reducing their diversity. The lower OTU richness found in SB-LC could be due to the shortness of period SB-ST-I, resulting in granules not mature enough to cope with 50 mg L^−1^ of amino acids compared to those communities of AB for this period. There were no statistical differences in the richness between reactors for a given period; hence, the modulation of the bacterial diversity was independent of the Aa feeding strategy. The lowest values of the Simpson index and, reciprocally, the highest of the Shannon index were found in the absence of Aa in both AGS-SBRs (Table SI.3). Subsequently, the presence of Aa independently of the concentration resulted in higher functional organizations of the AGS bacterial communities due to their lower richness and higher unevenly distribution in these samples (Marzorati et al., 2008).

**Fig. 4:**
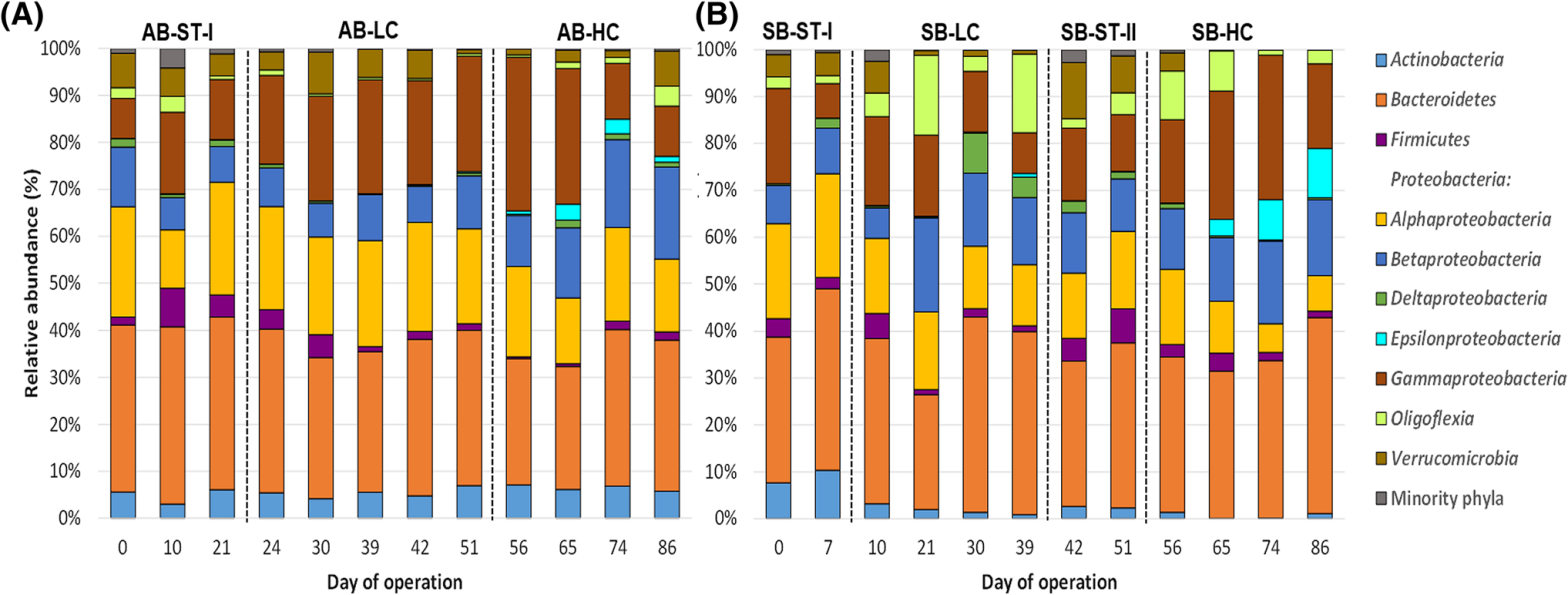
Average relative abundance of dominant fungal phyla (RA < 1%) identified by high-throughput Illumina sequencing from biomass samples retrieved from **A** acclimatization (*AB*), and **B** shock (SB) AGS-SBRs

The 1382 OTUs belonged to 23 different phyla; 5 of them were considered the majority phyla (RA > 1%). In addition, *Proteobacteria* was composed of 5 classes with RA > 1%, which were also regarded as main groups. Overall, the bacterial communities of both AGS-SBRs were mainly composed of *Bacteroidetes* (33.41%), *Gammaproteobacteria* (18.48%), *Alphaproteobacteria* (17.02%), *Betaproteobacteria* (12.45%), *Actinobacteria* (4.15%), *Verrucomicrobia* (4.18%), *Oligoflexia* (3.95%), *Firmicutes* (2.87%), *Deltaproteobacteria* (1.38%), and *Epsilonproteobacteria* (1.34%). The hotchpotch Minority phyla only accounted for 0.77%. The bacterial profiles at the phylum level among samples are shown in Fig. 4.

According to Table SI.4, the RAs of all the dominant bacterial groups were statistically different among periods for a given AGS-SBR, except *Verrucomicrobia* in AB and *Bacteroidetes* and *Deltaproteobacteria* in SB. In AB, the highest level of Aa (AB-HC stage) stimulated *Actinobacteria*, *Betaproteobacteria*, and *Gammaproteobacteria* and reduced RAs of *Alphaproteobacteria*, *Bacteroidetes Epsilonproteobacteria*, *Firmicutes*, and Minority phyla. Likewise, under similar conditions in SB-HC, *Epsilonproteobacteria* and *Oligoflexia* turned more abundant, and RAs of *Actinobacteria*, *Alphaproteobacteria*, *Firmicutes*, *Gammaproteobacteria*, *Verrucomicrobia*, and Minority phyla were inhibited. In addition, several significant differences were distinguished in the RAs of these dominant groups between the two AGS-SBRs for a given period. *Actinobacteria* was more abundant in AB-ST-I period compared with SB-ST-I. Regarding the differences between periods AB-LC and SB-LC, *Actinobacteria*, *Alphaproteobacteria*, and *Gammaproteobacteria* were enriched in AB and *Betaproteobacteria*, *Epsilonproteobacteria*, and *Oligoflexia* were more abundant in SB. Finally, whereas *Actinobacteria*, *Alphaproteobacteria* and *Verrucomicrobia* had higher RAs in period AB-HC than those of SB-HC, *Bacteroidetes* were over-represented in SB-HC compared with AB-HC. Therefore, different trends were observed in the dynamic of bacterial structures between reactors at the phylum-like level, suggesting that both the concentration of Aa and the addition strategy modulated the structures of the bacterial communities at the phylum level.

The main bacterial phyla were previously reported as dominant in other AGS systems (Liu et al., 2019c; Gao et al., 2020; Wei et al., 2020). However, an enrichment in *Bacteroidetes* was observed independently of the sample. Generally considered, *Bacteroidetes* specialized in degrading high molecular weight compounds and prefer growing attached to particles, as are AGS (Fernández-Gomez et al., 2013). Therefore, due to their proteolytic abilities, this phylum could have been an essential metabolizer of sulphur Aa. On the contrary, the phylum *Firmicutes* has been proposed as the leading anaerobic amino acid degrading group (Tang et al., 2005); however, this phylum was not enriched in any period or strategy condition, indicating Aa catabolism independent of these bacteria.

**Fig. 5:**
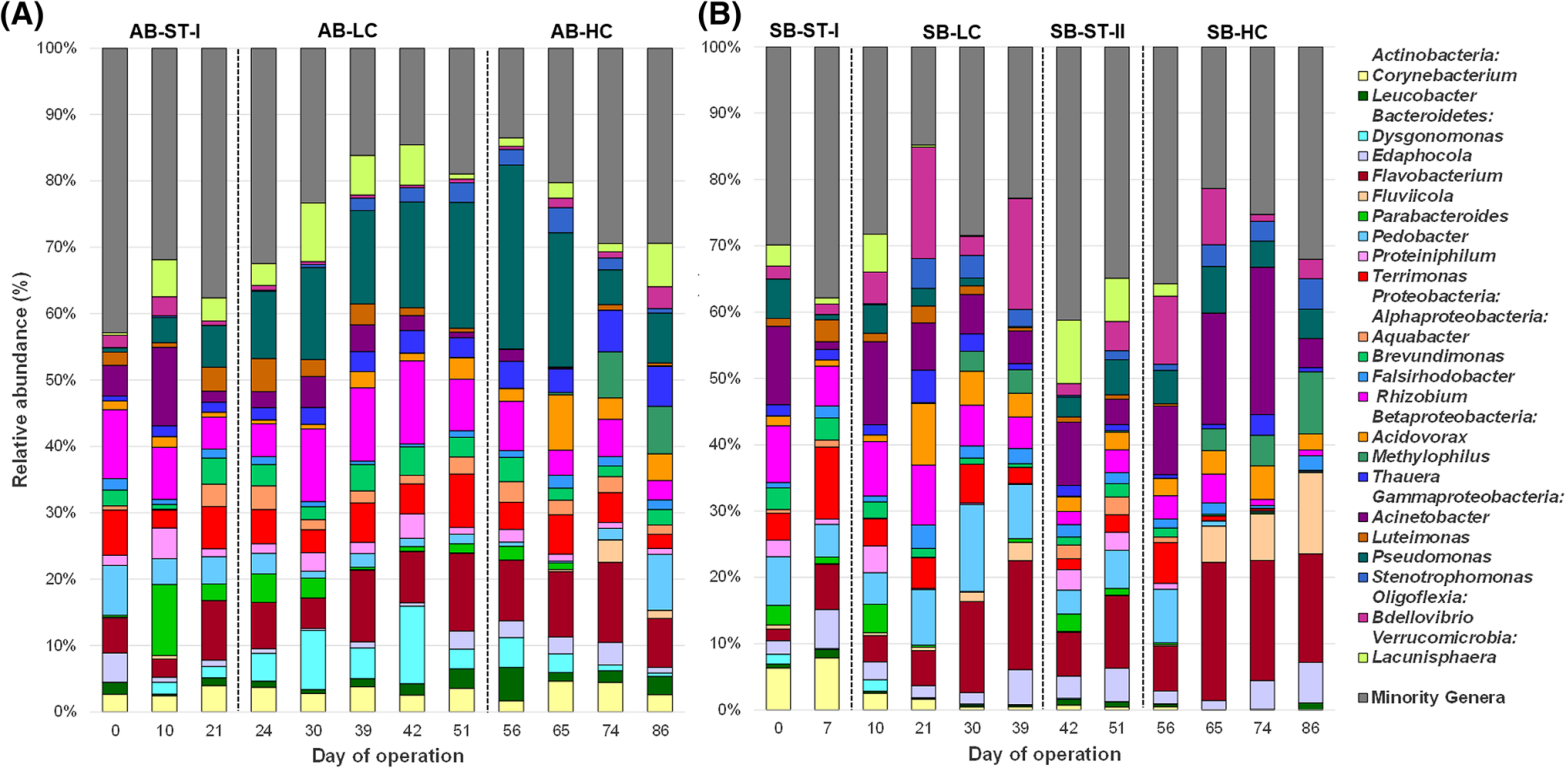
Average relative abundance of dominant bacterial OTUs (RA < 1%) identified by high-throughput Illumina sequencing from biomass samples retrieved from **A** acclimatization (*AB*), and **B** shock (*SB*) AGS-SBRs

The 1378 OTUs were distributed into 401 different genera, and 22 genera displayed RAs > 1% and were considered dominant genera. The distribution of the main genera among the samples is depicted in Fig. 5. According to their RA (sorted in decreasing order) they were the following: *Flavobacterium* (*Bacteroidetes*, 9.39%), *Pseudomonas* (*Gammaproteobacteria*, 7.98%), *Rhizobium* (*Alphaproteobacteria*, 6.17%), *Acinetobacter* (*Gammaproteobacteria*, 5.92%), *Terrimonas* (*Bacteroidetes*, 4.26%), *Pedobacter* (*Bacteroidetes*, 4.22%), *Bdellovibrio* (*Oligoflexia*, 3.73%), *Lacunisphaera* (*Verrucomicrobia*, 3.10%), *Acidovorax* (*Betaproteobacteria*, 2.96%), *Edaphocola* (*Bacteroidetes*, 2.53%), *Thauera* (*Betaproteobacteria*, 2.50%), *Corynebacterium* (*Actinobacteria*, 2.46%), *Dysgonomonas* (*Bacteroidetes*, 2.08%), *Brevundimonas* (*Alphaproteobacteria*, 2.04%), *Stenotrophomonas* (*Gammaproteobacteria*, 1.73%), *Methylophilus* (*Betaproteobacteria*, 1.62%), *Parabacteroides* (*Bacteroidetes*, 1.61%), *Proteiniphilum* (*Bacteroidetes*, 1.55%), *Fluviicola* (*Bacteroidetes*, 1.49%), *Falsirhodobacter* (*Alphaproteobacteria*, 1.43%), *Aquabacter* (*Alphaproteobacteria*, 1.34%), *Luteimonas* (*Gammaproteobacteria*, 1.32%), and *Leucobacter* (*Actinobacteria*, 1.16%). Altogether, these 22 genera represented more than 70% of the total high-quality sequences and the Minority genera accounting the remaining 30%. Several dominant genera agreed with the more abundant genera previously described in other AGS-SBRs (Henriet et al., 2016; Xu et al., 2018; Gao et al., 2020; He et al., 2021).

According to Table SI.5, there were several statistical differences after feeding with Aa-enrichment influent. In this sense, under 50 mg L^−1^ of Aa, in AB-LC*, Dysgonomonas*, *Pseudomonas*, *Stenotrophomonas*, and *Thauera* increased their RA genera compared to those of the no Aa addition; and, similarly, *Acidovorax*, *Bdellovibrio*, *Methylophilus*, and *Stenotrophomonas* were significantly increased in SB-LC. On the contrary, the genera whose RAs were reduced under this concentration were *Bdellovibrio*, *Falsirhodobacter*, *Pedobacter*, and Minority genera in AB-LC; and *Acinetobacter*, *Aquabacter*, *Brevundimonas*, *Leucobacter*, and Minority genera in SB-LC. Besides, the use of 100 mg L^−1^ of cysteine and 100 mg L^−1^ of methionine under the acclimatization strategy (AB-HC vs AB-LC) stimulated *Acidovorax*, *Bdellovibrio, Falsirhodobacter*, *Fluviicola*, *Leucobacter*, *Methylophilus*, *Thauera* and reduced the RAs of *Acinetobacter*, *Dysgonomonas*, *Luteimonas*, *Proteiniphilum*, and *Rhizobium*. Similarly, adding 100 mg L^−1^ of both Aa under the shock strategy (SB-HC vs SB-ST-II) increased the RAs of *Fluviicola* and *Stenotrophomonas* and reduced those of *Aquabacter*, *Brevundimonas*, *Corynebacterium*, *Lacunisphaera*, *Leucobacter*, *Luteimonas*, *Parabacteroides*, *Proteiniphilum* and Minority genera. Hence, the highest concentration of Aa clearly resulted in a shift in the bacterial structure of the dominant genera, as was previously reported by Gonzalez-Martinez et al. (2016) and Rodriguez-Sanchez et al. (2016) when they analyzed the effect of the addition of cysteine and methionine in anaerobic digesters, respectively. Regarding the differences among reactors for a given period, only *Corynebacterium* presented statistical differences between AB-ST-I and SB-ST-I; however, several differences were found between reactors when the influent was enriched in Aa. In this sense, 11 genera were increased, and other two genera were decreased in SB-HC compared to SB-ST-II. Hence, the shock strategy clearly inhibited the growth of a plethora of bacterial genera but allowed the proliferation of *Acinetobacter* and *Fluviicola*. *Acinetobacter* is often described in natural environments and harbours versatile metabolic capabilities such as degradation of several compounds; specifically, it can metabolize large amounts of organic matter in wastewater (Lang et al., 2019). Similarly, *Fluviicola* is distributed over a wide range of habitats, including wastewater, consuming several organic compounds encompassing proteins and Aa (Woyke et al., 2011). This suggests that the abrupt addition of 100 mg L^−1^ of Aa promoted the growth of both bacteria to cope with the stress situation caused by the high concentration of cysteine and methionine in the AGS-SBR in no acclimatized granules.

The heat map shown in Fig. SI.4 confirmed the divergences among periods in the dominant genera structure, which supports that the high addition of Aa modulated the bacterial structures of the AGS-SBRs. Also, the heatmap clearly displays that bacterial communities followed different trends according to the Aa concentration and addition strategy.

### Influence of the operational condition on the microbial communities

**Fig. 6:**
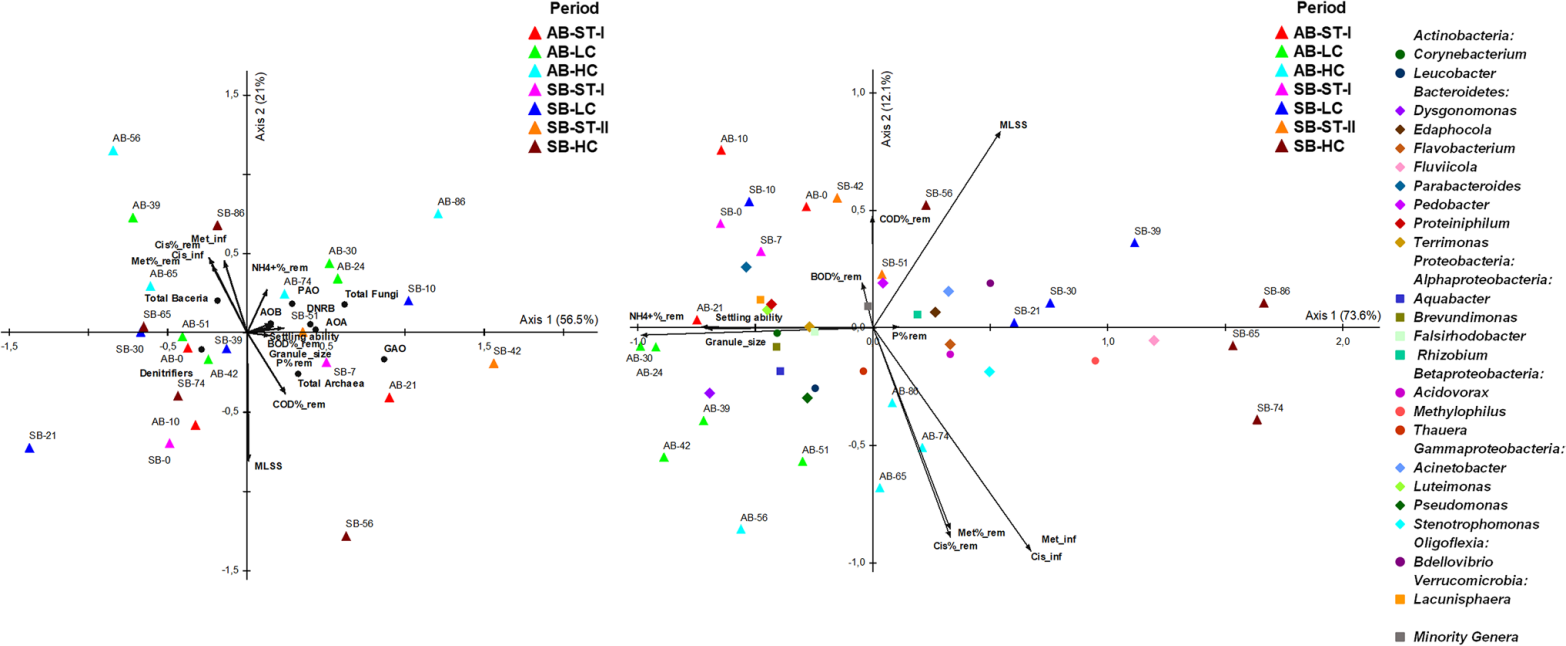
Nonmetric Multidimensional Scaling (NMS) ordination of **A** total abundances of the microbial abundances and **B** RAs of the dominant bacterial OTUs (RA > 1%) in biomass samples retrieved from biomass samples retrieved from AGS-SBRs, and their links with the operational parameters. COD%_rem: COD% removal, BOD%_rem: BOD5% removal, NH4 + %_rem: NH4 + % removal, P%_rem: P% removal, Cis%-rem: Cisteyne% removal, Met%-rem: Methionine% removal, Cis_int: [Cisteyne] of the influent, Met_inf: [Methionine] of the influent

An NMS analysis based upon the total abundance of the microbial groups and functional marker genes was carried out; also, the physicochemical properties and amino acid removal capabilities were linked to the NMS, aiming to deeper evaluate the effect of the abiotic variables on the size of the microbial communities. As a result, the NMS biplot (Fig. 6A) shows that the samples retrieved from both AGS-SBR were amply dispreaded over all the biplot space independently of the period and reactor. Therefore, neither the different Aa concentrations nor the application strategies were driving forces of the size of the bacterial communities. In addition, the vectors representing the abiotic data have short lengths, suggesting that different abiotic did not have substantial modulating capacities in the total abundances of the microbial groups. However, some strong correlations among the abiotic data and the total microbial abundances were found, as shown in Table SI.6. In this sense, the stimulating effect of the high presence of Aa on the size of the total bacterial populations and its negative impact on the size of total *Archaea* and GAO populations stand out. In this regard, the increases in the total bacterial abundances could be related to the maintenance of the nutrient removal efficiencies (Abzazou et al., 2018), which is necessary to increase due to its rise under the high Aa concentration.

Similarly, an NMS based on the RAs of the main bacterial genera plus the group of Minority Genera was also constructed (Fig. 6B). However, contrary to the observed for the NMS of the total microbial abundances (Fig. 6A), the different samples were clearly separated according to their corresponding operational period and reactor. This confirmed that the Aa concentration and the feeding strategy substantially impacted the bacterial community structure owing to these ordination patterns. In addition, the linking of the different operational parameters and the Aa removal into the NMS biplot resulted in longer vectors than those obtained for NMS of the microbial sizes. Accordingly, the structure of the bacterial community was strongly responsive to changes in these parameters. Besides, several strong correlations were found among the abiotic data and the RAs of the dominant genera (Table SI.7). Regarding the presence of high concentrations of cysteine and methionine and their removal rates, they were strong and positively related to *Acidovorax*, *Flavobacterium*, *Methylophilus*, *Stenotrophomonas*, and *Thauera* and presented significant negative correlations with the RAs of *Lacunisphaera*, *Parabacteroides*, *Pedobacter*, *Proteiniphilum*, and Minority genera. In this sense, the growing capacity of *Flavobacterium*, *Stenotrophomonas*, and *Thauera* using Aa as sole substrates has been previously defined as well as their extracellular proteolytic activities (Jankiewicz et al., 2016; Adhikari et al., 2017; Gulmus and Gormez, 2020; Isom et al., 2021). On the other hand, *Acidovorax* and *Methylophilus* have not been described as proteolytic or Aa-degrading bacterium (Burdman et al., 2005; Jin et al., 2019). Despite that the degradation of single sulphur Aa of these genera is not well-established, *Acidovorax*, *Flavobacterium*, *Methylophilus*, *Stenotrophomonas*, and *Thauera* are proposed as the prominent proteolytic genera in the AGS-SBRs, resulting in different metabolisms of cysteine and methionine during their removal. Strikingly, the protein-degrading *Proteiniphilum*, which can grow using several single Aa (Chen and Dong, 2005), was negatively related to the higher Aa removal, suggesting that cysteine or methionine degradation was independent of this bacterium. Finally, the genus *Clostridium* has been described as the main Aa-degrading microorganism through a Stickland reaction under anaerobic conditions; however, this genus was not dominant in the samples suggesting a cysteine and methionine catabolism independently of this bacterium.

To summarize, the presence of Aa resulted in intense pressure on the bacterial communities, deeply modulating the structures of the bacterial. Nevertheless, the Aa removal of the reactors was satisfactory, suggesting that AGS-SBR is a valuable technology in the WW treatment of influents enriched in sulphur Aa, inclusively when a shock strategy was used. However, the N removal was not enough efficiency. Therefore, further research is necessary to optimize the operational parameters to obtain a higher WW performance before scale-up in this process.

## Concluding remarks

High levels of cysteine and methionine negatively impacted the N removal; however, the elimination of the organic matter was generally satisfactory. In addition, an abrupt presence of both Aa further reduced N's removal performance.

Although methionine was more persistent than cysteine in the WW treatment, excellent removal rates of both Aa were observed independently of the feeding strategy, because removal rates below 80% were achieved for all scenarios, although previous acclimatization of microorganisms to Aa had a positive trend in comparison with microbial metabolism under shock stage.

The addition of 100 mg L^−1^ of cysteine and 100 mg L^−1^ of methionine had a low impact on the total abundance of the bacterial communities. However, the high presence of Aa sharply modulated the RAs of the dominant genera. Therefore, the structure of the bacterial community was more strongly responsive to the high concentrations of sulphur Aa. Similarly, the shock strategy more sharply modulates the bacterial structure than the absolute abundances of the different populations here determined.

*Acidovorax*, *Flavobacterium*, *Methylophilus*, *Stenotrophomonas*, and *Thauera* are proposed as the prominent proteolytic genera in the AGS-SBRs. Similarly, *Acinetobacter* and *Fluviicola* were important players to cope with the high presence of cysteine and methionine under the shock strategy.

Hence, the AGS-SBR technology is a valuable and suitable biological tool to treat WW enriched in sulphur Aa given the results obtained in this research.

## Supplementary Information

Below is the link to the electronic supplementary material.

Supplementary file1 (DOCX 54 KB)

Supplementary file2 (XLSX 462 KB)
