# Supplementary material for: Effects of sulphur amino acids on the size and structure of microbial communities of aerobic granular sludge bioreactors
Source: Amino Acids. 2022 May 25;54(10):1403–19. doi: 10.1007/s00726-022-03168-y (PMC9637606; doi:10.1007/s00726-022-03168-y)
Supplement: Supplementary file 1 — Supplementary file1 (DOCX 54 KB) [file 726_2022_3168_MOESM1_ESM.docx]

**Effects of sulphur amino acids on the size and structure of microbial communities of aerobic granular sludge bioreactors.**

Aurora Rosa-Masegosa^a,b 1^ (0000-0002-3540-8212), Lizandra Perez-Bou^b,c 1^ [(0000-0002-8597-7456), Barbara M](https://www.sciencedirect.com/science/article/pii/S2214714420306632?casa_token=2cCJwe8RQsYAAAAA:KrHKAuRtXqswWxpobk8Qzqon-Umrua-siuFDLdCVUADn8Txp5vlFddhLNTO1UNCdupqFwTrz#!)uñoz-Palazon^a,b *^ (0000-0002-0618-4213), Antonio Monteoliva-García^d^ (0000-0003-3663-2336), [Alejandro Gonzalez-Martinez](https://www.sciencedirect.com/science/article/pii/S2214714420306632?casa_token=2cCJwe8RQsYAAAAA:KrHKAuRtXqswWxpobk8Qzqon-Umrua-siuFDLdCVUADn8Txp5vlFddhLNTO1UNCdupqFwTrz#!)^a,b^ (0000-0002-4438-6430), [Jesus Gonzalez-Lopez](https://www.sciencedirect.com/science/article/pii/S2214714420306632?casa_token=2cCJwe8RQsYAAAAA:KrHKAuRtXqswWxpobk8Qzqon-Umrua-siuFDLdCVUADn8Txp5vlFddhLNTO1UNCdupqFwTrz#!)^a,b^ (0000-0003-0457-5453), David Correa-Galeote^a,b *^ (0000-0002-9776-8424).

*^a^ Microbiology Department, Faculty of Pharmacy, University of Granada, 18071 Granada, Andalucía, Spain*

*^b^* *Microbiology and Environmental Technology Section, Institute of Water Research, University of Granada, 18071 Granada, Andalucía, Spain*

*^c^ Microbial Biotechnology Group, Microbiology and Virology Department, Faculty of Biology, University of Habana, Cuba*

*^d^*  *Department of Civil Engineering, University of Granada, 18071 Granada, Spain*

^1^ Aurora Rosa-Masegosa and Lizandra Perez-Bou contributed equally to this article.

** Email: bmp@ugr.es; dcorrea@ugr.es*


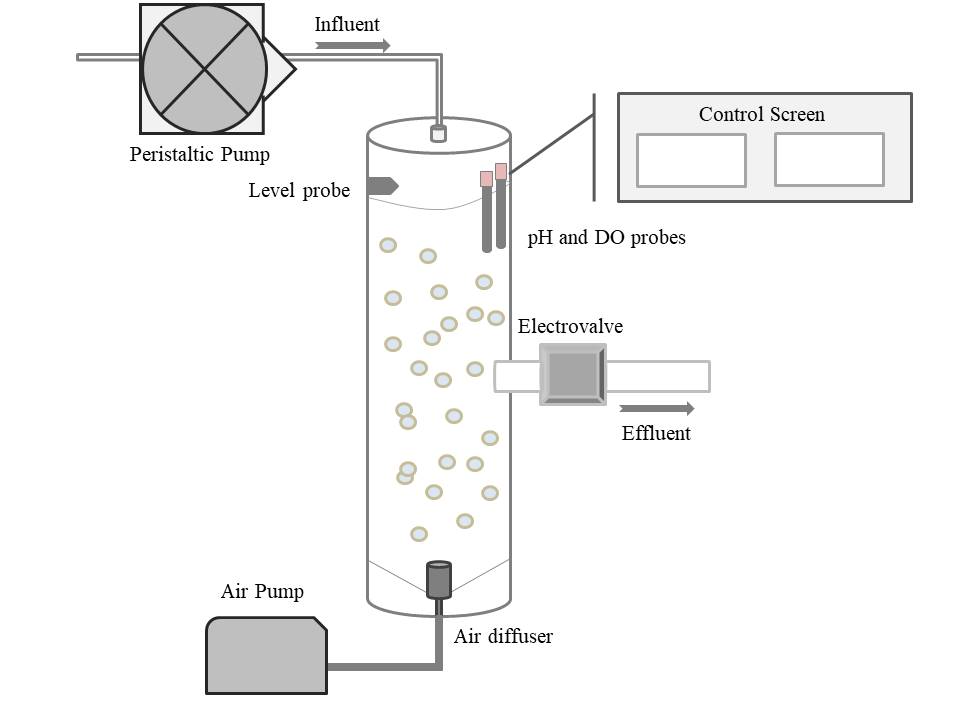


Figure S1.- Schematic diagram of the bioreactors employed for the experiments.
